# Microbial interaction between the succinate‐utilizing bacterium *Phascolarctobacterium faecium* and the gut commensal *Bacteroides thetaiotaomicron*

**DOI:** 10.1002/mbo3.1111

**Published:** 2020-08-28

**Authors:** Nao Ikeyama, Takumi Murakami, Atsushi Toyoda, Hiroshi Mori, Takao Iino, Moriya Ohkuma, Mitsuo Sakamoto

**Affiliations:** ^1^ Microbe Division/Japan Collection of Microorganisms RIKEN BioResource Research Center Tsukuba Ibaraki Japan; ^2^ Advanced Genomics Center National Institute of Genetics Mishima Shizuoka Japan; ^3^ PRIME Japan Agency for Medical Research and Development (AMED) Tsukuba Ibaraki Japan

**Keywords:** *Bacteroides thetaiotaomicron*, *Phascolarctobacterium faecium*, RNA‐Seq, succinate pathway, survival strategy

## Abstract

A large variety of microbes are present in the human gut, some of which are considered to interact with each other. Most of these interactions involve bacterial metabolites. *Phascolarctobacterium faecium* hardly uses carbohydrates for growth and instead uses succinate as a substrate. This study investigated the growth behavior of the co‐culture of the succinate‐specific utilizer *P*.* faecium* and the succinogenic gut commensal *Bacteroides thetaiotaomicron*. Succinate production by *B*.* thetaiotaomicron* supported the growth of *P*.* faecium* and concomitant propionate production via the succinate pathway. The succinate produced was completely converted to propionate. This result was comparable with the monoculture of *P*.* faecium* in the medium supplemented with 1% (w/v) succinate. We analyzed the transcriptional response (RNA‐Seq) between the mono‐ and co‐culture of *P*.* faecium* and *B*.* thetaiotaomicron*. Comparison of the expression levels of genes of *P*.* faecium* between the mono‐ and co‐cultured conditions highlighted that the genes putatively involved in the transportation of succinate were notably expressed under the co‐cultured conditions. Differential expression analysis showed that the presence of *P*.* faecium* induced changes in the *B*.* thetaiotaomicron* transcriptional pattern, for example, expression changes in the genes for vitamin B_12_ transporters and reduced expression of glutamate‐dependent acid resistance system‐related genes. Also, transcriptome analysis of *P*.* faecium* suggested that glutamate and succinate might be used as sources of succinyl‐CoA, an intermediate in the succinate pathway. This study revealed some survival strategies of asaccharolytic bacteria, such as *Phascolarctobacterium* spp., in the human gut.

## Graphical abstract

Succinate production by *Bacteroides thetaiotaomicron* supported the growth of *Phascolarctobacterium faecium* and concomitant propionate production via the succinate pathway. We analyzed the transcriptional response between mono‐ and co‐cultures of these two species. In *P*.* faecium*, the genes putatively involved in the transportation of succinate were notably expressed during co‐culture. Transcriptome analysis of *P*.* faecium* suggested that glutamate and succinate might be used as sources of succinyl‐CoA, an intermediate in the succinate pathway.

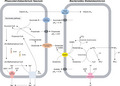

## 1 INTRODUCTION

Microbe–microbe interactions in the human gut have been increasingly recognized and analyzed in multidisciplinary fields. However, key factors in the interactions remain incompletely understood. Bacterial metabolites that include short‐chain fatty acids (SCFAs), such as acetate, propionate, and butyrate (succinate and lactate are considered SCFA precursors), are associated with human health and disease (Hosseini, Grootaert, Verstraete, & Van de Wiele, 2011; Koh, Vadder, Kovatcheva‐Datchary, & Bäckhed, 2016). Succinate and lactate are utilized by certain groups of anaerobic bacteria (Louis & Flint, 2017).

The microbiota of the human gut consists of a variety of microorganisms. Many are unclassified or uncultured anaerobic bacteria. During our attempts to recover new microbes from human feces, we observed bacteria that barely use carbohydrates for growth and instead use succinate as a substrate.

*Phascolarctobacterium faecium* is an obligately anaerobic and Gram‐negative bacterium that was first isolated from koala feces (Del Dot, Osawa, & Stackebrandt, 1993). Recently, it was reported that *P*.* faecium* abundantly colonizes the human gut (Wu et al., 2017). The functional role of *P*.* faecium* in the human gut is unknown. *P*.* faecium* utilizes succinate. It grows poorly on common blood agar, but adding succinate to the medium improves growth. In a previous study, we found that the *P*.* faecium* JCM 30894 genome lacked fumarate reductase, which is an enzyme that is necessary for the production of succinate (Ogata et al., 2019).

Among the human gut microbiota, the genus *Bacteroides* produces acetate and succinate as the main metabolites, so that a symbiotic relationship based on succinate is conceivable. Excess accumulation in the intestine of succinate induces diarrhea (Fernández‐Veledo & Vendrell, 2019; Ferreyra et al., 2014), and the presence of succinate‐utilizing bacteria may have beneficial effects on humans. *Bacteroides thetaiotaomicron* commonly inhabits the human gut and is capable of digesting polysaccharides (Flint, Bayer, Rincon, Lamed, & White, 2008; Porter, Luis, & Martens, 2018; Xu et al., 2003). *B*.* thetaiotaomicron* produces succinate as the main metabolite (Das, Ji, Kovatcheva‐Datchary, Bäckhed, & Nielsen, 2018).

To determine microbial interactions in the human gut, we used *P*.* faecium* and *B*.* thetaiotaomicron* as the model organisms. The co‐culture of *P*.* faecium* and *B*.* thetaiotaomicron* has not been previously studied. The trophic interaction between the mucin‐degrading bacterium *Akkermansia muciniphila* and the butyrate‐producing bacterium *Anaerostipes caccae* has been described (Chia et al., 2018). The authors demonstrated the use of metatranscriptomics (RNA‐Seq) as an explorative approach to study the expressional changes of *A*.* muciniphila* in response to *A*.* caccae*. We also used metatranscriptomics to explore the interaction of succinate‐producing and succinate‐utilizing bacteria from the human gut.

## 2 MATERIALS AND METHODS

### 2.1 Bacterial strains and growth conditions

*Phascolarctobacterium faecium* JCM 30894 and *B*.* thetaiotaomicron* JCM 5827^T^ were obtained from the Japan Collection of Microorganisms (JCM), RIKEN BioResource Research Center, Tsukuba, Japan. Normally, two strains were maintained on Eggerth Gagnon agar (Merck) supplemented with 5% (v/v) horse blood (EG; JCM Medium No. 14) for 2–4 days at 37°C in a gas atmosphere of H_2_, CO_2_, and N_2_ 1:1:8 (v/v) ratio.

### 2.2 Growth stimulation of *P*.* faecium* by *B*.* thetaiotaomicron*

*Bacteroides thetaiotaomicron* JCM 5827^T^ was streaked on one‐half of an EG plate using an inoculating loop. An inoculum of *P*.* faecium* JCM 30894 was similarly streaked on another half of the same EG plate. The monoculture of each strain was also performed as a control.

### 2.3 Growth stimulation of *P*.* faecium* by the addition of succinate

*Phascolarctobacterium faecium* JCM 30894 from a 6‐day plate culture were suspended in phosphate‐buffered saline (PBS). A 1% (v/v) suspension (MacFarland standard 3 turbidity) was inoculated into Gifu Anaerobic Medium Broth (GAM Broth, Nissui Pharmaceutical Co., Tokyo, Japan) that was not supplemented or supplemented with 1% (w/v) succinate (adjusted to pH 7.0). The broth was cultured experiments that were performed in anaerobic serum bottles sealed with butyl‐rubber stoppers at 37°C in an atmosphere of CO_2_ and N_2_ (1:9, v/v). Cultures were sampled at 0, 18, 20, 22, 24, 42, 44, 46, and 48 hr for analysis of metabolites and measurements of optical density at 660 nm (OD_660_). OD_660_ was measured using an Ultrospec 2100 *pro* spectrophotometer (Amersham Biosciences, Piscataway, NJ, USA). The pH of the medium was measured using a Twin pH compact pH meter (HORIBA, Kyoto, Japan).

### 2.4 Co‐culture

Co‐culture experiments were performed in GAM broth using anaerobic serum bottles sealed with butyl‐rubber stoppers at 37°C at the aforementioned culture conditions. *B*.* thetaiotaomicron* JCM 5827^T^ cells from a 2‐day plate culture were suspended in PBS. Suspensions (1% v/v; MacFarland standard 3) were added to GAM broth followed by 5 hr of incubation to allow accumulation of metabolites. A 1% (v/v) suspension (MacFarland standard 3) of *P*.* faecium* JCM 30894 was then added to the *B*.* thetaiotaomicron* cultures. Cultures were sampled at 0, 5, 23, 29, 47, and 53 hr for analysis of metabolites and measurements of OD_660_. For transcriptomic analysis, bacterial pellets received after 2 days of incubation were suspended in TRIzol Reagent (Life Technologies, Carlsbad, CA, USA) and stored at −20°C until used for RNA purification. A pure culture of each strain was also incubated for 2 days.

### 2.5 Analysis of metabolites

One milliliter of bacterial culture was centrifuged, and the supernatant was used for high‐performance liquid chromatography (HPLC) analysis. Metabolites were quantified using an HPLC system equipped with a model SPD‐M20A diode array detector model (Shimadzu, Kyoto, Japan) and a Rezex ROA‐Organic acid H^+^ (8%) column (Phenomenex, Torrance, CA, USA). The analytical conditions were as follows: eluent, 0.0025 N sulfuric acid; flow rate, 0.5 ml/min; detection, ultraviolet (UV) 210 nm; and column temperature 55°C. Succinate, propionate, and acetate were used as standards.

### 2.6 RNA purification

Total RNA was isolated by using the TRIzol Max Bacterial RNA Isolation Kit (Life Technologies) and the RNeasy Mini Kit (QIAGEN, Valencia, CA, USA) as described previously (Chia et al., 2018).

### 2.7 RNA sequencing

RNA samples with RNA Integrity Number ≥6.4 were used for the preparation of sequencing libraries. The libraries were constructed using two methods. One was a combination of MICROBExpress Bacterial mRNA Enrichment Kit (Ex) (Thermo Fisher Scientific, Waltham, MA, USA) and TruSeq Stranded mRNA Library Prep (Illumina, San Diego, CA, USA). The other method used the NEBNext rRNA Depletion Kit (Bacteria) (Nx) (New England Biolabs, Inc., Ipswich, MA, USA) and TruSeq Stranded mRNA Library Prep, according to the manufacturer's protocols. An Ex kit was used for the monoculture of each strain and co‐culture of two strains. An Nx kit was used for the monoculture of *B*.* thetaiotaomicron* and co‐culture. The final libraries were then sequenced on an Illumina HiSeq 2500 platform with 100 bp paired‐end sequencing reads.

### 2.8 Transcriptome analysis

Illumina adapter sequences and low‐quality bases were trimmed from raw fastq reads with fastp v0.20 (Chen, Zhou, Chen, & Gu, 2018). Forward and reverse reads were independently quality filtered, and qualified reads were combined into one fastq file. Qualified reads were then mapped on the RefSeq genomes of *B*. *thetaiotaomicron* VPI 5482^T^ (=JCM 5827^T^) (GCF_000011065.1) and *P*. *faecium* JCM 30894 (GCF_003945365.1) using BWA‐MEM v0.7.17 (Li & Durbin, 2009). The number of reads mapped within a protein‐coding sequence (CDS, including those annotated as pseudogenes) was counted using htseq‐count v0.11.2 (Anders, Pyl, & Huber, 2015) without MAPQ score filtering. *B*. *thetaiotaomicron* genes that were differentially expressed between the mono‐ and co‐cultured conditions were assessed using DESeq2 v1.26.0 (Love, Huber, & Andres, 2014). Genes with total read counts <10 were eliminated before DESeq2 analysis. Because of the difficulty in preparing a sufficient number of RNA samples from monocultured *P*. *faecium* for DESeq2 analysis, we instead normalized the read counts of *P*. *faecium* genes to transcript per million (TPM) values (Wagner, Kin, & Lynch, 2012) and compared the expression level of each gene among samples. To estimate the function of genes, we referred to the Kyoto Encyclopedia of Genes and Genomes (KEGG) Orthology database of the two strains (Kanehisa, Sato, Kawashima, Furumichi, & Tanabe, 2016) in addition to the RefSeq annotations. Fastq files obtained in this study have been deposited in the DNA Data Bank of Japan (DDBJ) under the accession numbers DRR228499–DRR228515.

## 3 RESULTS

### 3.1 Effect of *B*.* thetaiotaomicron* on the growth of *P*.* faecium*

**FIGURE 1:**
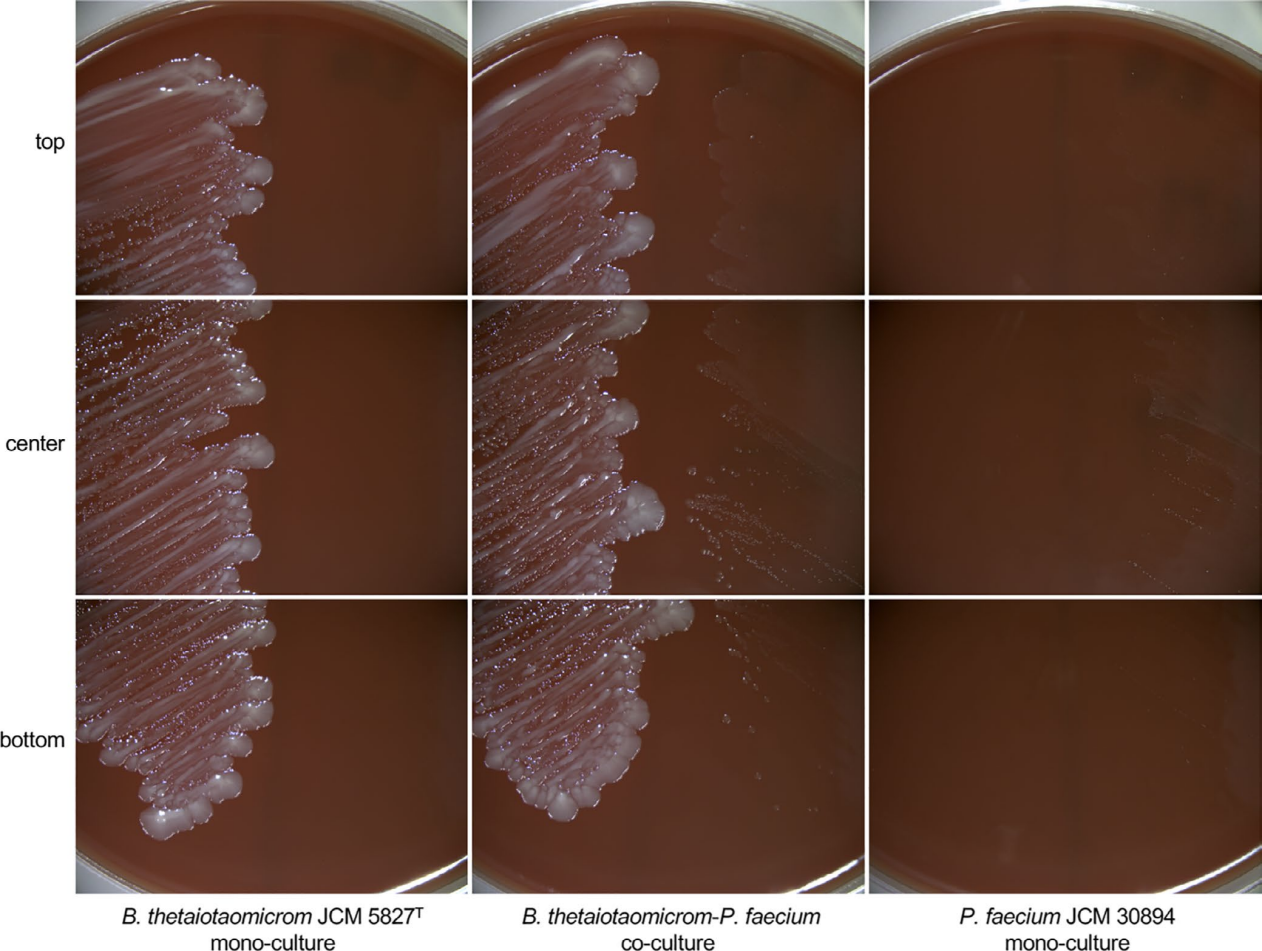
Growth stimulation of *Phascolarctobacterium faecium* by *Bacteroides thetaiotaomicron*. Strains were cultured on EG medium for 4 days at 37°C in an atmosphere of 1:1:8 H_2_/CO_2_/N_2_

*Phascolarctobacterium faecium *JCM 30894 monoculture formed small pinpoint colonies on the EG medium (Figure 1). Upon co‐culture with *B*.* thetaiotaomicron* JCM 5827^T^, *P*.* faecium* JCM 30894 grew well and formed slightly larger colonies compared with monoculture (especially around the colony border). There was no difference in the growth of *B*.* thetaiotaomicron* JCM 5827^T^ in mono‐ and co‐culture conditions with *P*.* faecium* JCM 30894.

### 3.2 Effect of succinate on the growth of *P*.* faecium*

**FIGURE 2:**
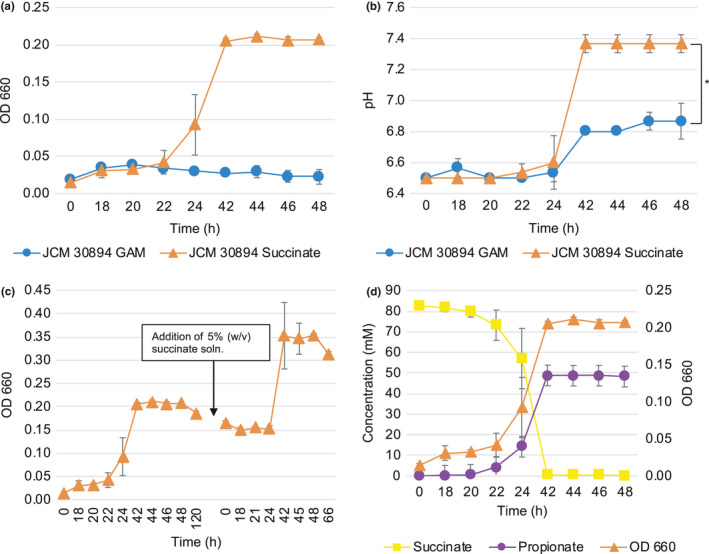
Growth stimulation of *Phascolarctobacterium faecium* by succinate. (a) *P*.* faecium* was inoculated into GAM broth that was not supplemented or supplemented with 1% (w/v) succinate. Experiments were performed in triplicate, and error bars represent the standard deviation between each biological replicate. (b) pH of the medium was measured at the time of sampling. Significant differences were determined by Student's *t* test. Significance was set at *p* < 0.05 (two‐tailed). * indicates significant differences, *p* < 0.01. (c) 5% (w/v) succinate solution (a final concentration of 1%) was added to *P*.* faecium* cultures at 120 hr. (d) The metabolite profile was analyzed by using HPLC

*Phascolarctobacterium faecium *JCM 30894 hardly grew in GAM broth with no evident turbidity. The addition of 1% (w/v) succinate significantly stimulated *P*.* faecium* growth (Figure 2a). This result agreed with previous observations (Ogata et al., 2019). *P*.* faecium* JCM 30894 began growing at approximately 22 hr. At 42 hr, the OD_660_ reached 0.200. The pH of the cultures without succinate was around 6.50–6.87, but that of cultures with succinate significantly increased to 7.37 after 42 hr with the growth of *P*.* faecium* JCM 30894 (*p* < 0.01, Figure 2b). Growth was stimulated further by the addition of succinate. After 120 hr, 5% (w/v) succinate solution (adjusted to pH 7.0) was added to *P*.* faecium* cultures to a final concentration of 1% (v/v). *P*.* faecium* JCM 30894 continued to grow (Figure 2c). At 42 hr (a total of 162 hr), the OD_660_ reached 0.352. In the presence of 1% (w/v) succinate, growth was observed with the consumption of succinate, and the production of propionate was detected (Figure 2d). Approximately 80 mM succinate was converted to 49 mM propionate.

### 3.3 Co‐culture of *P*.* faecium* and *B*.* thetaiotaomicron*

**FIGURE 3:**
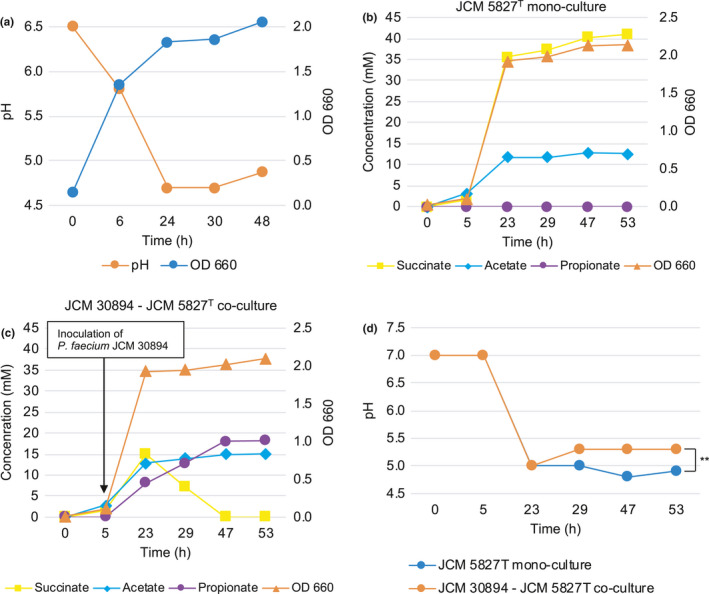
Co‐culture of *Phascolarctobacterium faecium* and *Bacteroides thetaiotaomicron*. (a) The pH and growth profile of *B*.* thetaiotaomicron* were investigated in a preliminary experiment. Results of (b) monoculture and (c) co‐culture. *B*.* thetaiotaomicron* was inoculated at 0 h followed by *P*.* faecium* at 5 hr. (d) The pH of monoculture and co‐culture was measured at the time of sampling. **significant differences *p* < 0.05

**FIGURE 4:**
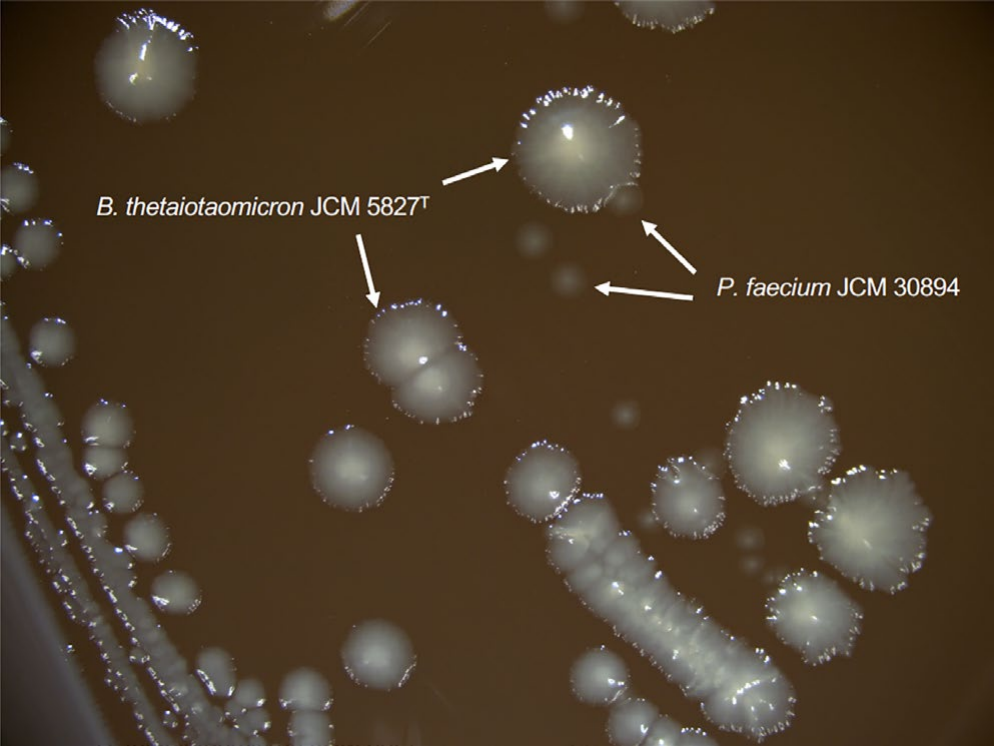
Colonies of *Phascolarctobacterium faecium* and *Bacteroides thetaiotaomicron* in co‐culture

In a preliminary experiment, the growth of *B*.* thetaiotaomicron* JCM 5827^T^ was investigated (Figure 3a). Based on these results, *P*.* faecium* JCM 30894 was inoculated into a 5‐hour culture of *B*.* thetaiotaomicron* JCM 5827^T^ (Figure 3c). *B*.* thetaiotaomicron* JCM 5827^T^ grown as monoculture produced 41 mM succinate and 12.6 mM acetate, but no propionate was detected (Figure 3b). On the other hand, *P*.* faecium* and *B*.* thetaiotaomicron* co‐culture produced propionate in addition to succinate and acetate (Figure 3c). In the co‐culture, up to 15.0 mM succinate was detected after 23 hr of incubation. As the amount of propionate was increased, the amount of succinate decreased and was not detected after 47 hr. Alternatively, 18.2 mM propionate was detected. This result was comparable with the monoculture of *P*.* faecium* JCM 30894 in GAM broth supplemented with 1% (w/v) succinate (Figure 2d). No significant differences in growth were observed between the mono‐ and co‐culture of *P*.* faecium* and *B*.* thetaiotaomicron* (Figure 3b,c). However, as in the succinate‐amended culture described above (Figure 2b), there was a significant difference (*p* < 0.05) in the final pH of the monoculture (pH 4.9) and co‐culture (pH 5.3; Figure 3d). At this point, it was unclear whether the growth of *P*.* faecium* JCM 30894 was promoted or not. To assess this, a portion of the co‐culture was plated onto the EG medium. *P*.* faecium* JCM 30894 formed small colonies near the large colonies of *B*.* thetaiotaomicron* JCM 5827^T^ (Figure 4). The colonies of *P*.* faecium* JCM 30894 were 1 to 2 mm in diameter and larger than those (0.1–0.2 mm) of the monoculture of this strain on the EG medium (Figure 1).

### 3.4 Transcriptomes of co‐culture of *P*.* faecium* and *B*.* thetaiotaomicron*

On average, 20 million reads were generated per sample, which is above the recommended sequence depth of 5–10 million reads for a single bacterial transcriptome (Haas, Chin, Nusbaum, Birren, & Livny, 2012). The Nx kit enabled the removal of more rRNA. The number of reads mapped to the CDSs was higher. In the co‐culture sample, most of the reads were derived from *B*.* thetaiotaomicron*. An average of 9.78% (Ex kit) and 56.7% (Nx kit) of reads were mapped on the CDSs of *B*.* thetaiotaomicron*, while the percentages of *P*.* faecium* CDS‐mapped read count were average 0.04% (Ex kit) and 0.3% (Nx kit; Table A1).

### 3.5 Highly expressed genes of *P*.* faecium* co‐cultured with *B*.* thetaiotaomicron*

The genes involved in the succinate pathway were expressed in mono‐ and co‐culture. However, due to the insufficient number of reads, it was difficult to determine whether there were significant changes in the expression levels. In the co‐culture, two genes encoding SLC13/DASS family transporters (PFJ30894_RS03075 and PFJ30894_RS04475) exhibited much larger TPM values compared to the monoculture. Furthermore, sodium/glutamate symporter (PFJ30894_RS00375), Glu/Leu/Phe/Val dehydrogenase (PFJ30894_RS04940), and a gene cluster consisting of PFJ30894_RS01150 (4Fe‐4S dicluster domain‐containing protein), PFJ30894_RS01155 (2‐oxoacid:acceptor oxidoreductase subunit alpha), PFJ30894_RS01160 (2‐oxoacid:ferredoxin oxidoreductase subunit beta), and PFJ30894_RS01165 (pyruvate/ketoisovalerate oxidoreductase gamma subunit) were highly expressed in the co‐culture (Table A2). Moreover, genes encoding chaperones and stress response factors also exhibited larger TPM values in the co‐culture than in the monoculture (Table 1).

**TABLE 1:** Transcript per million (TPM) value of genes encoding chaperones and stress response factors in *P*.* faecium*

Locus tag	Strand	RefSeq annotation	KEGG Orthology	MoEx TMP	CoEx TPM	CoNx TPM
PFJ30894_RS00455	−	Bacteriocin family protein	–	4076.86	**9225.09**	**13,777.01**
PFJ30894_RS00630	+	Universal stress protein	–	1066.11	**3010.79**	**4517.97**
PFJ30894_RS06505	+	Heat‐inducible transcription repressor HrcA	K03705	3021.14	**8547.96**	**13,627.11**
PFJ30894_RS06510	+	Nucleotide exchange factor GrpE	K03687	4062.34	**8751.30**	4761.36
PFJ30894_RS06515	+	Molecular chaperone DnaK	K04043	923.46	**2476.38**	**5102.12**
PFJ30894_RS06520	+	Molecular chaperone DnaJ	K03686	324.39	**1099.11**	**2476.24**
PFJ30894_RS03240	−	Universal stress protein	–	418.42	**53,544.17**	**80,951.83**
PFJ30894_RS07940	+	Recombinase RecA	K03553	815.27	**6812.12**	**10,200.12**
PFJ30894_RS07945	+	Hypothetical protein	K03565	305.71	**4065.23**	**6452.08**
PFJ30894_RS11160	−	Chaperonin GroEL	K04077	914.48	**4093.71**	**5986.45**
PFJ30894_RS11165	−	Co‐chaperone GroES	K04078	1842.30	**4516.56**	**8424.61**

Note

Bold type indicates TPM values ≥2 in the co‐culture (CoEx, CoNx) as compared with the monoculture (MoEx).

### 3.6 Differential expression between *B*.* thetaiotaomicron* in monoculture and co‐culture with *P*.* faecium*

The genome of *B*.* thetaiotaomicron* possesses 4794 CDSs, of which 4786 (99.8%) were expressed in at least one sample. We used an adjusted *p*‐value <0.05 as the cutoff for significantly regulated genes. Genes with the same direction of variation between the Ex and Nx kits were defined as “consistent change” and those with a different direction of variation as “inconsistent change.” A total of 1495 genes were consistent change, of which 538 were log_2_ fold change (FC) ≥1 in both kits. In the co‐culture with *P*.* faecium*, a gene cluster consisting of BT_RS13000 (glutamate decarboxylase), BT_RS13005 (glutaminase A), BT_RS13010 (two‐pore domain potassium channel family protein), and BT_RS13015 (glutamate:GABA antiporter *gadC*) was downregulated in *B*.* thetaiotaomicron*. In contrast, a gene cluster for an ATP‐binding cassette (ABC) transporter consisting of BT_RS02750 (HlyD family efflux transporter periplasmic adaptor subunit), BT_RS02755 (ATP‐binding cassette domain‐containing protein), BT_RS02760 (ABC transporter permease), and BT_RS02765 (ABC transporter permease) was upregulated in the co‐culture (Table A3). Another gene set for an ABC transporter (BT_RS10625, BT_RS10630, and BT_RS10635) and neighboring BT_RS10605 (TonB‐dependent receptor plug domain‐containing protein) were also upregulated in the co‐culture. Degnan, Barry, Mok, Taga, and Goodman (2014) identified that these genes encode BtuCDF (an ABC transporter) and BtuB (an outer membrane transporter, designated as *btuB3*), transporters for vitamin B_12_ and its analogous corrinoids. Two other gene loci that encode BtuB (*btuB1* and *btuB2*) in the *B*. *thetaiotaomicron* genome and another gene set for BtuCDF were consecutively coded with *btuB2*. *ButB1* (BT_RS07540) was also upregulated in the co‐culture, although the adjusted *p*‐value in the Ex kit was slightly larger than 0.05. In contrast, *btuB2* (BT_RS09905) was downregulated in the co‐culture (Table 2). Besides, a cluster of genes encoding subunits of ATP synthase was upregulated in the monoculture (Table 3).

**TABLE 2:** Differential expression of genes for transporters of vitamin B_12_ in *B*.* thetaiotaomicron*.

Locus tag	Strand	RefSeq annotation	KEGG Orthology	Ex log_2_FC	Nx log_2_FC	Ex adj*P*	Nx adj*P*
BT_RS07540	+	TonB‐dependent receptor	–	0.53	0.7	6.49E‐02	6.14E‐09
BT_RS09890	−	ABC transporter ATP‐binding protein	K02013	1.22	−0.43	6.88E‐02	1.61E‐01
BT_RS09895	−	Iron ABC transporter permease	K02015	0.4	−1.4	3.35E‐01	5.82E‐24
BT_RS09900	−	ABC transporter substrate‐binding protein	K02016	−0.03	−0.64	9.20E‐01	9.07E‐05
BT_RS09905	−	TonB‐dependent receptor plug domain‐containing protein	K02014	−0.81	−1.36	3.16E‐08	4.00E‐38
BT_RS10605	−	TonB‐dependent receptor plug domain‐containing protein	K02014	0.68	0.79	2.32E‐03	2.50E‐12
BT_RS10625	+	ABC transporter substrate‐binding protein	K02016	1.01	0.69	1.60E‐02	7.91E‐05
BT_RS10630	+	Iron ABC transporter permease	K02015	1.48	0.63	6.09E‐03	5.90E‐04
BT_RS10635	+	ABC transporter ATP‐binding protein	K02013	1.95	1.58	2.24E‐04	3.10E‐14

Note

Negative values indicate upregulation in the monoculture, and positive values indicate upregulation in the co‐culture.

**TABLE 3:** Differential expression of genes encoding subunits of ATP synthase in *B*.* thetaiotaomicron*

Locus tag	Strand	RefSeq annotation	KEGG Orthology	Ex log_2_FC	Nx log_2_FC	Ex adj*P*	Nx adj*P*
BT_RS03560	+	F0F1 ATP synthase subunit beta	K02112	−1.77	−0.97	3.21E‐135	1.26E‐22
BT_RS03565	+	ATP synthase F1 subunit epsilon	K02114	−1.62	0.15	1.23E‐53	6.43E‐01
BT_RS03570	+	Hypothetical protein	–	−0.87	−1.32	1.14E‐04	5.89E‐16
BT_RS03575	+	F0F1 ATP synthase subunit A	K02108	−0.86	−0.54	8.84E‐16	3.40E‐06
BT_RS03580	+	ATP synthase F0 subunit C	K02110	−0.52	−0.10	2.00E‐02	3.14E‐01
BT_RS03585	+	F0F1 ATP synthase subunit B	K02109	−0.82	−0.45	2.63E‐11	1.29E‐02
BT_RS03590	+	F0F1 ATP synthase subunit delta	K02113	−0.46	−0.44	1.52E‐03	1.00E‐02
BT_RS03595	+	F0F1 ATP synthase subunit alpha	K02111	−0.51	−0.14	9.44E‐07	2.97E‐01
BT_RS03600	+	F0F1 ATP synthase subunit gamma	K02115	−0.77	−0.50	4.52E‐12	1.70E‐04

Note

Negative values indicate upregulation in the monoculture, and the positive value indicates upregulation in the co‐culture.

## 4 DISCUSSION

In this study, we investigated the growth behavior of the co‐cultured *P*.* faecium* and *B*.* thetaiotaomicron*. The p*K*_a_ values for succinate and propionate are 4.16 and 4.87, respectively. The pH of the culture supplemented with succinate increased from 6.6 to 7.4 after 42 hr. As inferred from the above‐mentioned p*K*_a_ values for each organic acid, this is consistent with a decrease in succinate and increase in propionate. The pH of the co‐culture also increased slightly from 5.0 to 5.3 after 29 hr (Figure 3d). The collective results support the view that the pH change is associated with the conversion of succinate to propionate.

The genus *Phascolarctobacterium* contains three known species (including *P*.* faecium*). *Phascolarctobacterium succinatutens* was isolated from human feces (Watanabe, Nagai, & Morotomi, 2012). *Phascolarctobacterium wakonense* was isolated from common marmoset feces (Shigeno, Kitahara, Shime, & Benno, 2019). These two species, as well as *P*.* faecium*, grew well in medium supplemented with succinate. This finding may be one of the characteristics of this genus. In the human gut, *Phascolarctobacterium* spp. convert succinate to propionate, which is a health‐promoting microbial metabolite (Hosseini et al., 2011).

The three different biochemical pathways for propionate production include the succinate, acrylate, and propanediol pathways (Reichardt et al., 2014; Vidra & Németh, 2018). *Bacteroides* spp. possess the succinate pathway (Macy, Ljungdahl, & Gottschalk, 1978; Reichardt et al., 2014). This pathway is also present in *Phascolarctobacterium* spp. (Ogata et al., 2019; Reichardt et al., 2014). However, due to the lack of fumarate reductase, it is presumed that *P*.* faecium* JCM 30894 is unable to produce succinate, a key metabolite of the succinate pathway (Ogata et al., 2019). Therefore, co‐existence with succinate‐producing bacteria, such as *Bacteroides*, is essential for *Phascolarctobacterium* spp. to inhabit the human gut and produce propionate. Although *Bacteroides* spp. can convert succinate to propionate by the succinate pathway, it has been reported that succinate accumulates in cultures of *Bacteroides fragilis* under growth conditions where phosphoenolpyruvate carboxykinase is repressed at high CO_2_ partial pressures and high dilution rates (Caspari & Macy, 1983). Furthermore, one of the conversion reactions from succinate to propionate involves methylmalonyl‐CoA mutase, which requires vitamin B_12_ (Louis & Flint, 2017). Succinate accumulates in B_12_‐depleted cultures of *Prevotella ruminicola* (Strobel, 1992). The genomes of *Bacteroides* spp. and *Phascolarctobacterium* spp. have been assessed for the presence of biosynthesis pathways for eight B vitamins: biotin, cobalamin (vitamin B_12_), folate, niacin, pantothenate, pyridoxine, riboflavin, and thiamin (Magnúsdóttir, Ravcheev, de Crécy‐Lagard, & Thiele, 2015; Ogata et al., 2019). *B*.* thetaiotaomicron* VPI 5482^T^ (=JCM 5827^T^) lacked the upstream genes required for vitamin B_12_ biosynthesis. On the other hand, *Phascolarctobacterium* spp. (*P*.* faecium* JCM 30894 and *P*.* succinatutens* YIT 12067^T^) were predicted to possess a complete vitamin B_12_ biosynthesis pathway. Therefore, when grown as a monoculture, *B*.* thetaiotaomicron* JCM 5827^T^ probably accumulated succinate, but not propionate. In contrast, in the co‐culture of two species, *P*.* faecium* JCM 30894 can convert succinate to propionate, as this strain harbors the vitamin B_12_ biosynthesis pathway. Although *B*. *thetaiotaomicron* does not have complete gene sets for vitamin B_12_ biosynthesis, it instead harbors three genes (*btuB1–3*) encoding transporters for vitamin B_12_ and its analogous corrinoids. These genes are essential for the colonization of *B*. *thetaiotaomicron* in the intestine of germ‐free mice (Degnan et al., 2014). Interestingly, in the co‐culture, *btuB1* and *btuB3* were upregulated, but *btuB2* was downregulated. Degnan et al. (2014) reported that these three transporters exhibited different preferences among several corrinoid species. These results suggest that *B*.* thetaiotaomicron* changes the expression patterns of these transporter genes in response to the corrinoids provided by *P*. *faecium*. Previous experiments observed in the presence of vitamin B_12_ suggested that propionate production was associated with the conservation of biologically useful energy (Strobel, 1992). *P*.* faecium* may exist in an energy‐limited environment, and maximizing energy conservation during the production of propionate may be one strategy this bacterium uses to survive in the human gut. The conversion of the energy of the decarboxylation reaction into sodium ion (Na^+^) gradients by methylmalonyl‐CoA decarboxylase is the biological use of decarboxylation energy (Dimroth, 1987; Hilpert & Dimroth, 1982). The central energy conservation step in *Propionigenium modestum* (Schink & Pfennig, 1982) is the conversion of the energy of methylmalonyl‐CoA decarboxylation into a Na^+^ gradient, which in turn drives ATP synthesis via Na^+^‐activated ATPase (Dimroth, 1997; Dimroth & Schink, 1998; Hilpert, Schink, & Dimroth, 1984).

In this study, although the difference in the expression of the genes involved in the succinate pathway of *P*.* faecium* was not clear, high expressions of two genes encoding SLC13/DASS family transporter were observed in the co‐culture. One of the genes (PFJ30894_RS03075) is encoded consecutively with the gene cluster of the succinate pathway. The SLC13 transporter is part of the divalent anion:Na^+^ symporter (DASS) family (Mulligan, Fitzgerald, Wang, & Mindell, 2014). VcINDY, an SLC13 homologue from *Vibrio cholerae*, couples a Na^+^ gradient to the transport of succinate, a C_4_‐dicarboxylate (Mulligan et al., 2014). Therefore, it seems that *P*.* faecium* upregulated the SLC13 transporter to transport succinate produced by *B*.* thetaiotaomicron* into the cell.

It has been suggested that lower pH achieved in the co‐culture of *B*.* thetaiotaomicron* and *Bifidobacterium adolescentis* could potentially slow the growth and metabolism of *B*.* thetaiotaomicron* (Das et al., 2018). Furthermore, it has been reported that *B*.* thetaiotaomicron* DSM 2079^T^ (=JCM 5827^T^) showed a growth rate at pH 5.5 of approximately 40% of the growth at pH 6.7 (Duncan, Louis, Thomson, & Flint, 2009). As mentioned above, the pH of the co‐culture of *P*.* faecium* and *B*.* thetaiotaomicron* decreased from 7.0 to 5.0, but then increased to 5.3. Increased pH in co‐culture would improve the growth of *B*.* thetaiotaomicron*. Consequently, the co‐existence of these two species seems to be beneficial for each species. In the presence of *P*.* faecium*, *B*.* thetaiotaomicron* downregulated glutamate‐dependent acid resistance system‐related genes involved in glutaminase A and glutamate decarboxylase activity and the antiporter GadC. Strategies adopted to face acid encounters include amino acid‐dependent systems (Lu et al., 2013; Pennacchietti, D’Alonzo, Freddi, Occhialini, & De Biase, 2018). In particular, the glutamate‐dependent acid resistance system is extremely powerful. *B*.* thetaiotaomicron* possesses this system (Pennacchietti et al., 2018). The difference in pH values between the monoculture (pH 4.9) and co‐culture (pH 5.3) resulted in the downregulation of the glutamate‐dependent acid resistance system‐related genes in the co‐culture. On the other hand, co‐cultured *P*.* faecium* highly expressed genes for chaperones and stress response factors. *P*.* faecium* did not grow in the monoculture but could grow in the co‐culture. Active transcription of chaperone genes could be associated with acid stress caused by succinate and acetate produced in the co‐culture. The molecular mechanisms adopted by Gram‐positive and Gram‐negative bacteria for coping with acid stress have been reviewed (Lund, Tramonti, & De Biase, 2014). ATPase (ATP synthase) is involved in acid resistance for *Escherichia coli* (Sun, Fukamachi, Saito, & Kobayashi, 2011, 2012), *Salmonella typhimurium* (Foster & Hall, 1990, 1991), and *Streptococcus faecalis* (Kobayashi, Suzuki, & Umemoto, 1986). In this study, *B*.* thetaiotaomicron* upregulated ATP synthase genes in the monoculture. This result is consistent with that of the aforementioned glutamate‐dependent acid resistance system, which is one of the mechanisms of protection against acid stress.

*B*.* thetaiotaomicron* may release compounds including glutamate and ammonium into the medium in response to acidic stress. Therefore, it is conceivable that *P*.* faecium* also transports glutamate into the cell using the Na^+^ gradient formed during succinate metabolism. The transported glutamate is converted to 2‐oxoglutarate by glutamate dehydrogenase, and 2‐oxoglutarate is converted to succinyl‐CoA by 2‐oxoglutarate oxidoreductase. As mentioned above, high expression of these enzyme genes was observed in the co‐culture with *B*.* thetaiotaomicron*, so that glutamate and succinate might be used as sources of succinyl‐CoA, an intermediate in the succinate pathway.

In the presence of *P*.* faecium*, *B*.* thetaiotaomicron* upregulated a gene set for an ABC transporter, putatively acting as an efflux pump. It has been reported that *B*.* fragilis* may induce efflux pump gene expression when encountered with secreted antibiotics or other potentially toxic components by competing with surrounding organisms (Ghotaslou, Yekani, & Memar, 2018). Thus, *B*.* thetaiotaomicron* may have expressed a defense mechanism in the presence of *P*.* faecium*.

Co‐culture experiments of *P*.* succinatutens* and the xylan‐utilizing and succinate‐producing bacterium *Paraprevotella xylaniphila* have also been reported (Watanabe et al., 2012). In the co‐culture, the numbers of *P*.* succinatutens* cells increase and succinate is converted to propionate. These findings may indicate one of the survival strategies of asaccharolytic *Phascolarctobacterium* spp. in the human gut. This idea is supported by the greater abundance of *Phascolarctobacterium* along with the increased abundance of *Bacteroides* in rats fed a high‐fat diet (Lecomte et al., 2015). The abundance of *B*.* fragilis* and *Bacteroides vulgatus* was positively correlated with both changes in body weight and fat mass. A previous study demonstrated that *P*.* faecium* colonizes the human gut in early life and develops to a high level in healthy adults, followed by a decrease in elderly individuals (Wu et al., 2017). The authors described that elderly individuals and those <1 year of age consumed relatively less fat and had a relatively low body weight. As inferred from rat experiments described above, this may result in a decrease in *Bacteroides* and the decrease in the available succinate for *P*.* faecium*.

**FIGURE 5:**
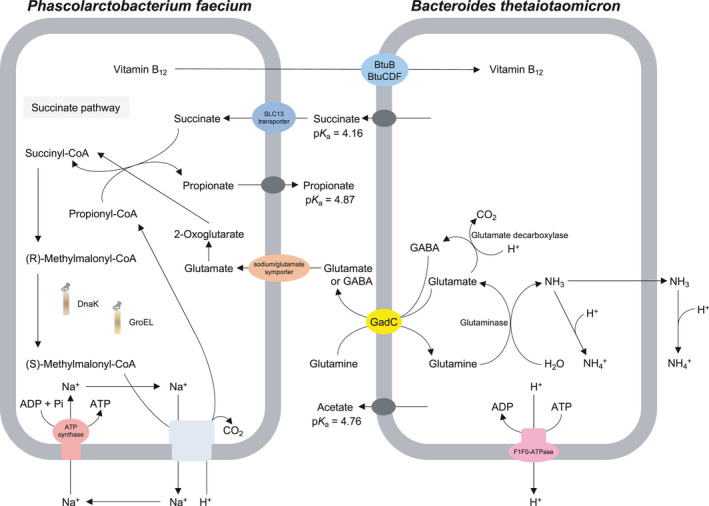
Schematic representation of *Phascolarctobacterium faecium* and *Bacteroides thetaiotaomicron* in co‐culture

In conclusion, we reveal some survival strategies of asaccharolytic bacteria, such as *Phascolarctobacterium* spp., in the human gut. The encounter between *P*.* faecium* and *B*.* thetaiotaomicron* in the human gut may result in a beneficial conversion of succinate to propionate. An overview of the microbial interaction between the succinate‐utilizing bacterium *P*.* faecium* and the gut commensal *B*.* thetaiotaomicron* is shown in Figure 5.

## CONFLICT OF INTEREST

None declared.

## AUTHOR CONTRIBUTIONS

**Nao Ikeyama:** Formal analysis (lead); Investigation (lead); Writing‐original draft (lead); Writing‐review & editing (lead). **Takumi Murakami:** Formal analysis (equal); Writing‐original draft (equal). **Atsushi Toyoda:** Funding acquisition (equal); Investigation (equal); Writing‐original draft (equal). **Hiroshi Mori:** Formal analysis (equal); Funding acquisition (equal); Writing‐original draft (equal). **Takao Iino:** Formal analysis (equal); Writing‐review & editing (equal). **Moriya Ohkuma:** Funding acquisition (equal); Writing‐review & editing (equal). **Mitsuo Sakamoto:** Conceptualization (lead); Formal analysis (lead); Funding acquisition (lead); Investigation (lead); Project administration (lead); Resources (lead); Supervision (lead); Validation (lead); Visualization (lead); Writing‐original draft (lead); Writing‐review & editing (lead).

## ETHICS STATEMENT

None required.

## Data Availability

The datasets used and analyzed during the current study are included in this published article.

## Appendix 1

**TABLE A1:** The number of reads mapped on the protein‐coding sequences (CDSs)

Sample	Total read count	*B*. *thetaiotaomicron* (4794 CDSs)		*P*. *faecium* (2277 CDSs)	
		CDS‐mapped read count	Read‐mapped CDS count	CDS‐mapped read count	Read‐mapped CDS count
Bt_A_Ex	17,156,707	655,289 (3.82)	4113 (85.79)	–	–
Bt_B_Ex	18,043,654	805,156 (4.46)	4238 (88.40)	–	–
Bt_C_Ex	17,306,996	762,024 (4.40)	4206 (87.73)	–	–
Bt_D_Ex	20,798,562	1,055,905 (5.08)	4350 (90.74)	–	–
Pf_Ex	16,883,728	–	–	1,027,297 (6.08)	2277 (100)
Co_A_Ex	17,945,740	1,587,043 (8.84)	4769 (99.48)	6377 (0.04)	574 (25.21)
Co_B_Ex	16,255,269	1,705,601 (10.49)	4759 (99.27)	7205 (0.04)	637 (27.98)
Co_C_Ex	18,441,787	1,731,455 (9.39)	4785 (99.81)	6836 (0.04)	628 (27.58)
Co_D_Ex	17,332,280	1,803,122 (10.40)	4777 (99.65)	7031 (0.04)	695 (30.52)
Bt_A_Nx	24,059,327	11,871,401 (49.34)	4746 (99.00)	–	–
Bt_B_Nx	23,968,675	11,706,125 (48.84)	4747 (99.02)	–	–
Bt_C_Nx	23,567,147	12,057,528 (51.16)	4749 (99.06)	–	–
Bt_D_Nx	23,261,196	10,812,167 (46.48)	4726 (98.58)	–	–
Co_A_Nx	23,624,293	15,116,851 (63.99)	4758 (99.25)	85,823 (0.36)	1361 (59.77)
Co_B_Nx	23,804,062	12,425,946 (52.20)	4747 (99.02)	66,204 (0.28)	1353 (59.42)
Co_C_Nx	23,020,409	13,891,365 (60.34)	4755 (99.19)	77,322 (0.34)	1363 (59.86)
Co_D_Nx	22,196,464	11,162,589 (50.29)	4740 (98.87)	58,955 (0.27)	1298 (57.00)

Note

Percentage of mapped reads and CDSs are indicated in parentheses.

Bt: *B*. *thetaiotaomicron*, Pf: *P*. *faecium*, Co: Co‐culture, Ex: MICROBExpress Bacterial mRNA Enrichment Kit, Nx: NEBNext rRNA Depletion Kit.

**TABLE A2:** Transcript per million (TPM) value of the genes of *Phascolarctobacterium faecium*.

Locus tag	Strand	RefSeq annotation	KEGG Orthology	MoEx TPM	CoEx TPM (average)	CoNx TPM (average)
Genes involved in the succinate pathway						
PFJ30894_RS01590	+	Cobalamin B_12_‐binding domain‐containing protein	K01849	295.72	0	0
PFJ30894_RS01595	+	Methylmalonyl‐CoA carboxyltransferase	K01604	338.18	0	0
PFJ30894_RS03730	+	Succinate CoA transferase	K18118	333.54	60.91	32.15
PFJ30894_RS03735	+	Methylmalonyl‐CoA mutase	K01848	224.08	0	31.48
PFJ30894_RS04255	+	Methylmalonyl‐CoA mutase	K01847	207.62	0	0
PFJ30894_RS04260	+	Methylmalonyl‐CoA mutase	K01847	234.07	0	0
PFJ30894_RS04410	+	Acetyl‐CoA hydrolase/transferase family protein	K18118	1104.25	525.48	989.68
PFJ30894_RS04415	+	Methylmalonyl‐CoA mutase family protein	K01848	1474.25	762.62	774.18
PFJ30894_RS04420	+	Cobalamin B_12_‐binding domain‐containing protein	K01849	765.35	169.18	671.45
PFJ30894_RS04430	+	Methylmalonyl‐CoA epimerase	K05606	534.06	209.5	448.4
PFJ30894_RS04435	+	Methylmalonyl‐CoA carboxyltransferase	K01604	356.74	0	91.02
PFJ30894_RS04440	+	OadG family protein	K23352	228.87	67.9	115.87
PFJ30894_RS04445	+	Biotin/lipoyl‐binding protein	K23351	551.21	121.18	128.31
PFJ30894_RS04450	+	Sodium ion‐translocating decarboxylase subunit beta	K20509	277.59	279.98	242.45
PFJ30894_RS04455	+	Methylmalonyl‐CoA carboxyltransferase	K01604	311.37	14.59	82.38
PFJ30894_RS04460	+	Sodium pump decarboxylase	K23352	138.97	0	0
PFJ30894_RS04465	+	Biotin/lipoyl‐binding protein	K23351	141.67	0	0
PFJ30894_RS04470	+	Sodium ion‐translocating decarboxylase subunit beta	K20509	157.25	0	15.65
Genes for SLC13 family transporters						
PFJ30894_RS03075	+	Citrate transporter	K14445	253.98	**2764.97**	**7569.79**
PFJ30894_RS04475	+	SLC13/DASS family transporter	K14445	1135.71	996.63	**3053.47**
Genes involved in the glutamate metabolism						
PFJ30894_RS00375	−	Sodium/glutamate symporter	K03312	439.74	**955.46**	**2475.13**
PFJ30894_RS01150	+	4Fe‐4S dicluster domain‐containing protein	K00176	359.17	357.32	501.28
PFJ30894_RS01155	+	2‐oxoacid:acceptor oxidoreductase subunit alpha	K00174	335.97	426.04	**689.65**
PFJ30894_RS01160	+	2‐oxoacid:ferredoxin oxidoreductase subunit beta	K00175	279.4	305.7	**651.11**
PFJ30894_RS01165	+	Pyruvate/ketoisovalerate oxidoreductase gamma subunit	K00177	276.8	**634.28**	**915.82**
PFJ30894_RS04940	+	Glu/Leu/Phe/Val dehydrogenase	K00260	2622.01	**6570.4**	**13,455.59**

Note

Bold type indicates TPM values ≥2 in the co‐culture (CoEx, CoNx) as compared with the monoculture (MoEx).

**TABLE A3:** Differential expression of genes of *Bacteroides thetaiotaomicron* between mono‐ and co‐cultured conditions

Locus tag	Strand	RefSeq annotation	KEGG Orthology	Ex log2FC	Nx log2FC	Ex adj*P*	Nx adj*P*
Genes for the glutamine/glutamate‐dependent acid resistance system							
BT_RS13000	+	Glutamate decarboxylase	K01580	−1.2	−1.15	7.04E‐50	1.28E‐12
BT_RS13005	+	Glutaminase A	K01425	−1.99	−1.95	1.04E‐120	2.22E‐28
BT_RS13010	−	Two‐pore domain potassium channel family protein	–	−1.72	−1.32	2.42E‐18	3.05E‐29
BT_RS13015	+	Amino acid permease	K20265	−0.92	−1.32	7.01E‐06	1.13E‐18
Genes for a putative efflux ABC transporter							
BT_RS02750	+	HlyD family efflux transporter periplasmic adaptor subunit	K01993	0.3	3.25	1.98E‐01	7.87E‐113
BT_RS02755	+	ATP‐binding cassette domain‐containing protein	K01990	2.5	5.23	1.13E‐29	0
BT_RS02760	+	ABC transporter permease	K01992	6.68	6.66	2.97E‐112	0
BT_RS02765	+	ABC transporter permease	K01992	3.21	4.14	5.49E‐178	0

Note

Negative values indicate upregulation in the monoculture, and positive values indicate upregulation in the co‐culture.
